# Suilysin remodels the cytoskeletons of human brain microvascular endothelial cells by activating RhoA and Rac1 GTPase

**DOI:** 10.1007/s13238-014-0037-0

**Published:** 2014-03-19

**Authors:** Qingyu Lv, Huaijie Hao, Lili Bi, Yuling Zheng, Xuyu Zhou, Yongqiang Jiang

**Affiliations:** 1 State Key Laboratory of Pathogen and Biosecurity, Institute of Microbiology and Epidemiology, Academy of Military Medical Sciences, Beijing, 100071 China; 2 CAS Key Laboratory of Pathogenic Microbiology and Immunology, Institute of Microbiology, Chinese Academy of Sciences, Beijing, 100101 China

**Dear Editor,**

*Streptococcus suis* (*S. suis*) is a Gram-positive, facultative anaerobic coccus and an important emerging pathogen. It is associated with bacterial meningitis in adults, especially in southeastern Asia (Wertheim et al., 2009). A striking feature of *S. suis* infection is the resulting complications, which may include deafness and vestibular dysfunction. Complications of some kind affect 50% of *S. suis* patients in Europe and 73% of those in Asia (Gottschalk et al., 2010). Some *S. suis* surface-associated factors, such as the capsule and Fhb, may affect the pathogenesis of meningitis. Of these secreted factors, suilysin was found to be the most important. It has been found to be toxic to various types of cells (Lalonde et al., 2000). It is also involved in modulation of the interactions of *S. suis* with different host cells (Charland et al., 2000; Segura et al., 2006). Epidemic *S. suis* ST7 strains were found to produce more suilysin than other ST strains (unpublished data). This has been shown to contribute to their ability to travel across the epithelial barrier, which they do in a TLR4-dependent manner. This ability is also associated with the increased severity of *S. suis* infection (unpublished data). Recently, subcytolytic suilysin was shown to promote *S. suis* association with epithelial cells without causing the formation of functional (cytolytic) pores. This indicated that sublytic concentrations of suilysin also contributed to pathogenesis by modification of host-pathogen interactions (Seitz et al., 2013). However, the mechanism underlying suilysin-mediated modulation of microbial-host interactions has not yet been fully explained.

In the present study, human brain microvascular endothelial cells (hBMECs) were treated with non-cytotoxic and sublytic concentrations of the culture supernatant of *S. suis* serotype 2 strain 05ZYH33 and stained with FITC-labeled phalloidin at different time points after challenging the culture with sublytic supernatant. Fluorescence microscopy showed stress fibers, lamellipodia, and filopodia to be visible 5–40 min after treatment (Fig. S1). These results demonstrated that sublytic concentrations of *S. suis* culture supernatant rearranged the cytoskeletons of the hBMECs. Suilysin has been shown to be the most important virulence factor in the secreted supernatant of *S. suis*. Suilysin is presumed to be the effective component of *S. suis* culture supernatant, and the factor primarily responsible for the remodeling of hBMEC cytoskeletons. To confirm this, hBMEC cells were treated with sublytic concentrations of suilysin protein (0.3 μg/mL) purified from *S. suis* cell culture supernatant over different periods of time. As shown in Fig. 1, suilysin led to the rapid formation of filopodia, stress fibers, and lamellipodia in hBMECs. Lipids and cholesterol have been identified as the receptors of suilysin, as well as some cholesterol-dependent cytolysins (CDCs). To confirm the cholesterol dependence of the effects of suilysin, suilysin was exposed to cholesterol at mass ratios of 1:1 and 1:5 for 15 min at 37°C immediately before it was applied to the hBMEC cells for 10 min. As shown in Fig. 1, pretreatment of suilysin with cholesterol at a 1:1 ratio reduced the formation of lamellipodia and filopodia, although actin stress fibers still formed. When suilysin was pretreated with cholesterol at a 5:1 cholesterol:suilysin ratio, the suilysin-induced changes in actin were completely absent. The changes in actin were also absent when cellular cholesterol was removed using 2 mmol/L methyl-β-cyclodextrin (MβCD) to inhibit suilysin binding. Taken together, these results indicate that the changes in actin cytoskeleton organization induced by suilysin were cholesterol-dependent.

**Figure 1:**
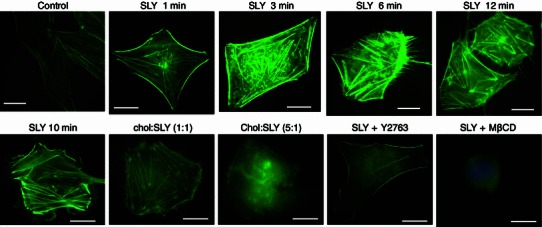
**Suilysin-induced changes in the organization of the actin cytoskeleton were dependent on the concentrations of cholesterol and GTPase**. hBMEC cells were treated with sublytic concentrations of suilysin protein for indicated periods after pretreatment regimens. Actin filaments were stained with FITC-labeled phalloidin. Chol: cholesterol. Y27632: ROCK inhibitor. MβCD: cellular cholesterol depletion

Generally, Rho-subclass GTPases produce stress fibers, Rac produces filopodia and lamellipodia, and Cdc42 produces filopodia (Tapon and Hall, 1997). To confirm the effects of suilysin on these pathways, RhoA-associated kinase (ROCK) inhibitor Y27632 was used to pretreat hBMECs for 1 h before suilysin challenge. The inhibitor Y27632 prevented the formation of stress fibers after 10 min of suilysin treatment (Fig. 1). However, RhoA, which is upstream of ROCK in the signaling pathway, was still activated (Fig. 2E). These results indicated that the changes in actin cytoskeleton organization induced by suilysin were GTPase-dependent. Next, the molecular basis of the changes in actin induced by *S. suis* culture supernatant was assessed. A rhotekin-based pull-down assay showed approximately 3-fold activation of RhoA 5 min after treatment by sublytic concentration of *S. suis* culture supernatant, which peaked at 10 min and was found to have returned to resting levels at 30 min (Fig. 2A). A p21-activated, kinase-based pull-down assay showed that Rac1 peaked at 10 min, and its activation showed a downward trend that remained constant for 20 min (Fig. 2B). Then, the activation of RhoA and Rac1 in hBMECs after suilysin treatment was assessed. A rhotekin-based pull-down assay showed RhoA to be activated from 10 min to 30 min after suilysin treatment, after which it decreased, reaching resting levels by 45 min (Fig. 2C). A p21-activated kinase-based pull-down assay showed that Rac1 was activated at 10 min after suilysin treatment and peaked at 20 min (Fig. 2D). Taken together, these results suggest that *S. suis* culture supernatant and suilysin protein can activate RhoA and Rac1 in a time-dependent fashion, but their activation effects peak at different times.

**Figure 2:**
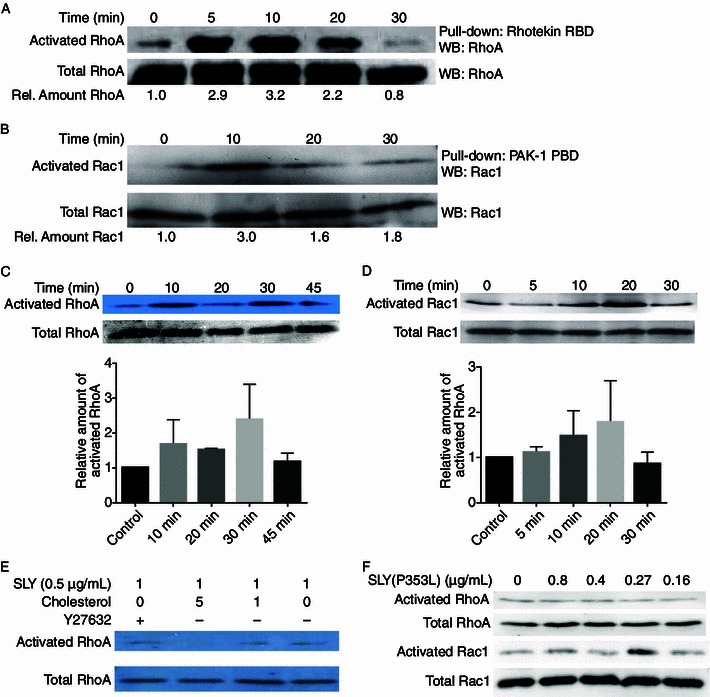
**Suilysin-induced activation of GTPases in hBMECs was dependent on the concentration of cholesterol but not hemolytic activity**. hBMEC cells were serum-starved and then treated with (A and B) *S. suis* supernatant or (C and D) suilysin protein at sublytic concentrations for indicated periods. (E) hBMEC cells pretreated with Y27632 or left untreated were then treated with suilysin that had been pretreated with cholesterol or left untreated. (F) hBMEC cells were treated with P353L mutant of suilysin at indicated concentrations. (A, C, E and F) RhoA and (B, D and F) Rac1 activation was detected using pull-down and Western blot assays with total RhoA and Rac1 amounts at each point in time as shown in the images of the endogenous control and representative samples. (C and D) Relative amounts of activated RhoA and Rac1 from three independent experiments with mean ± SEM (bottom panel)

Because the suilysin-induced changes in the organization of the actin cytoskeleton were found to be cholesterol-dependent, the issue of whether the suilysin-induced activation of RhoA is also cholesterol-dependent was addressed. Suilysin was pretreated with cholesterol at mass ratios of 1:1 and 1:5 for 15 min at 37°C. Then, the hBMEC cells were treated with this suilysin-cholesterol mixture for 10 min. As shown in Fig. 2E, suilysin pretreated with cholesterol at a 1:1 ratio still activated RhoA. When suilysin was pretreated with cholesterol at a 5:1 cholesterol: suilysin ratio, the activation of RhoA induced by suilysin was completely inhibited. Previous studies have shown that hemolysin-positive *S. suis* strains used adherence and suilysin-induced BMEC injury to move from the circulatory system to the central nervous system (Charland et al., 2000). This indicates that hemolytic activity is the basis for suilysin activity. However, in the present work, sublytic concentrations of suilysin were found to activate RhoA and Rac1, causing remodeling of the actin cytoskeleton. This indicated that suilysin might have functions independent of hemolytic activity. To further analyze the correlation between the GTPase activation induced by suilysin and the hemolytic activity of suilysin, a recombinant suilysin mutant, SLY (P353L), which was proven to lack hemolytic ability, was constructed (Xu et al., 2010). Different concentrations of purified SLY (P353L) protein were used to treat hBMECs for 10 min, and active GTPases were analyzed using pull-down assays. As shown in Fig. 2F, SLY (P353L), cannot activate RhoA, but can activate Rac1 at relatively low concentrations (0.27 μg/mL). These results suggested that the ability of suilysin to activate Rac1 was independent of its hemolytic activity.

Pathogenic microbes subvert normal host cell processes to create a specialized niche, facilitating their survival. Pathogens often target the cytoskeleton of the host cell, which they use for attachment, entry into cells, movement within and between cells, vacuole formation and remodeling, and avoidance of pathocytosis (Barbieri et al., 2002; Rottner et al., 2005). In this work, non-cytotoxic and sublytic concentrations of *S. suis* suilysin were found to remodel the actin cytoskeleton. These results contribute to understanding of the manner by which *S. suis* traverses the human blood-brain barrier (BBB). Recent studies also have shown that microbial translocation of the BBB involves host cell actin cytoskeletal rearrangements (Kim, 2006). For example, human BMEC actin cytoskeleton rearrangements were shown to be a prerequisite for human BMEC invasion by *E. coli* K1, group B *streptococcus*, and *L. monocytogenes* (Kim, 2006). In addition to invasion, some pathogens, such as *Neisseria meningococcus*, induce changes in the host cell cytoskeleton, recruiting endothelial cell adhesion molecules such as VE-cadherin to cortical plaques. This recruitment leads to the formation of ectopic intercellular junctional domains at the site of bacteria-host cell interaction, depletion of junctional proteins at the cell-cell interface, opening of intercellular junctions, and crossing the BBB in a paracellular manner (Coureuil et al., 2009; Coureuil et al., 2010). For this reason, actin rearrangement induced by suilysin might initiate the paracellular traversal of *S. suis* across the human BBB.

Several signal transduction pathways have been shown to be involved in rearranging the actin cytoskeleton. These include the FAK, PI3K, Src kinase, and Rho GTPase pathways. Among the members of the CDC group, pneumolysin is the only known pore-forming toxin that uses the small GTPases found in intact cells to alter the actin cytoskeleton (Iliev et al., 2007; Hupp et al., 2013). The present results demonstrate another case, showing that actin cytoskeleton rearrangment is a novel function of suilysin. These findings contribute to the current understanding of the mechanism by which *S. suis* traverses the human BBB, suggesting new potential targets for the treatment of meningitis.

## Electronic supplementary material

Below is the link to the electronic supplementary material.

Supplementary material 1 (PDF 565 kb)
