# Supplementary material for: Suilysin remodels the cytoskeletons of human brain microvascular endothelial cells by activating RhoA and Rac1 GTPase
Source: Protein Cell. 2014 Mar 19;5(4):261–4. doi: 10.1007/s13238-014-0037-0 (PMC3978165; doi:10.1007/s13238-014-0037-0)
Supplement: Supplementary file 1 — Supplementary material 1 (PDF 565 kb) [file 13238_2014_37_MOESM1_ESM.pdf]

Supplementary Materials

Suilysin remodels the cytoskeletons of human brain microvascular endothelial cells by activating RhoA and Rac1 GTPase

Supplemental figures S1–S2

Supplemental materials and methods

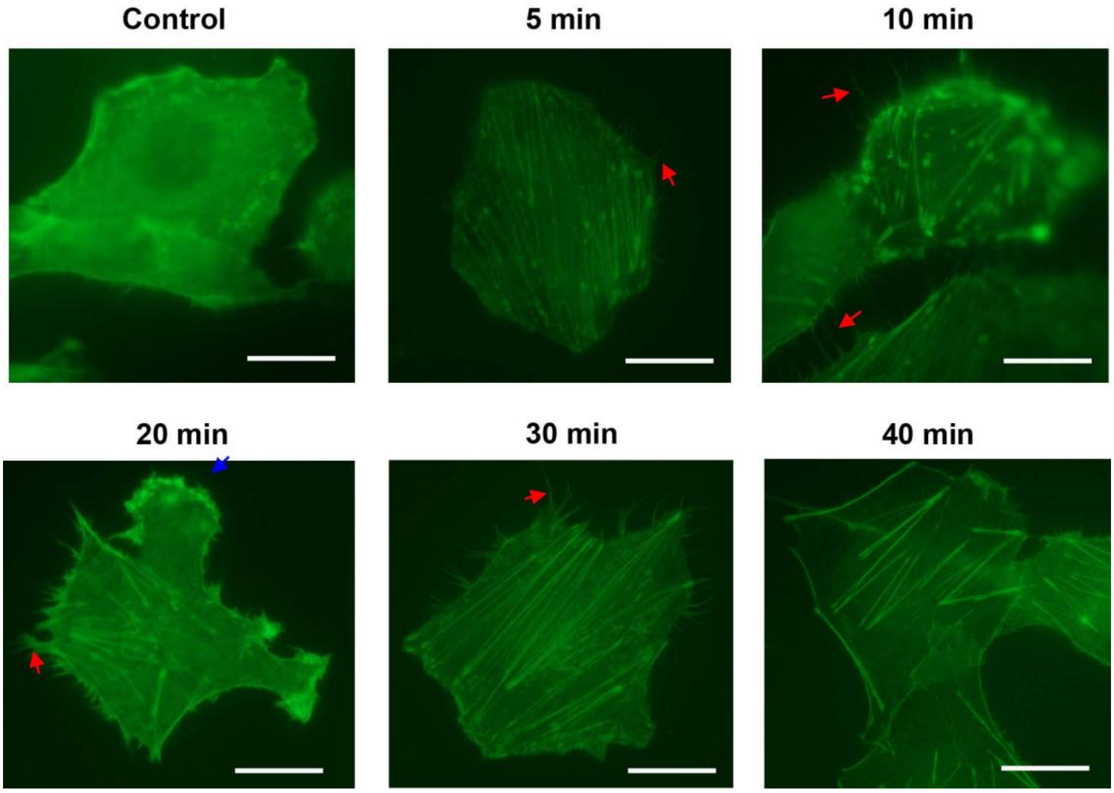

Figure S1. Analysis of *S. Suis* culture supernatant-induced changes in the organization of the actin cytoskeletons of hBMEC cells. Cells were treated with sublytic concentrations of *S. suis* culture supernatant for the indicated periods. Actin filaments were stained with FITC-labeled phalloidin; results showed the formation of filopodia, lamellipodia, and stress fibers.

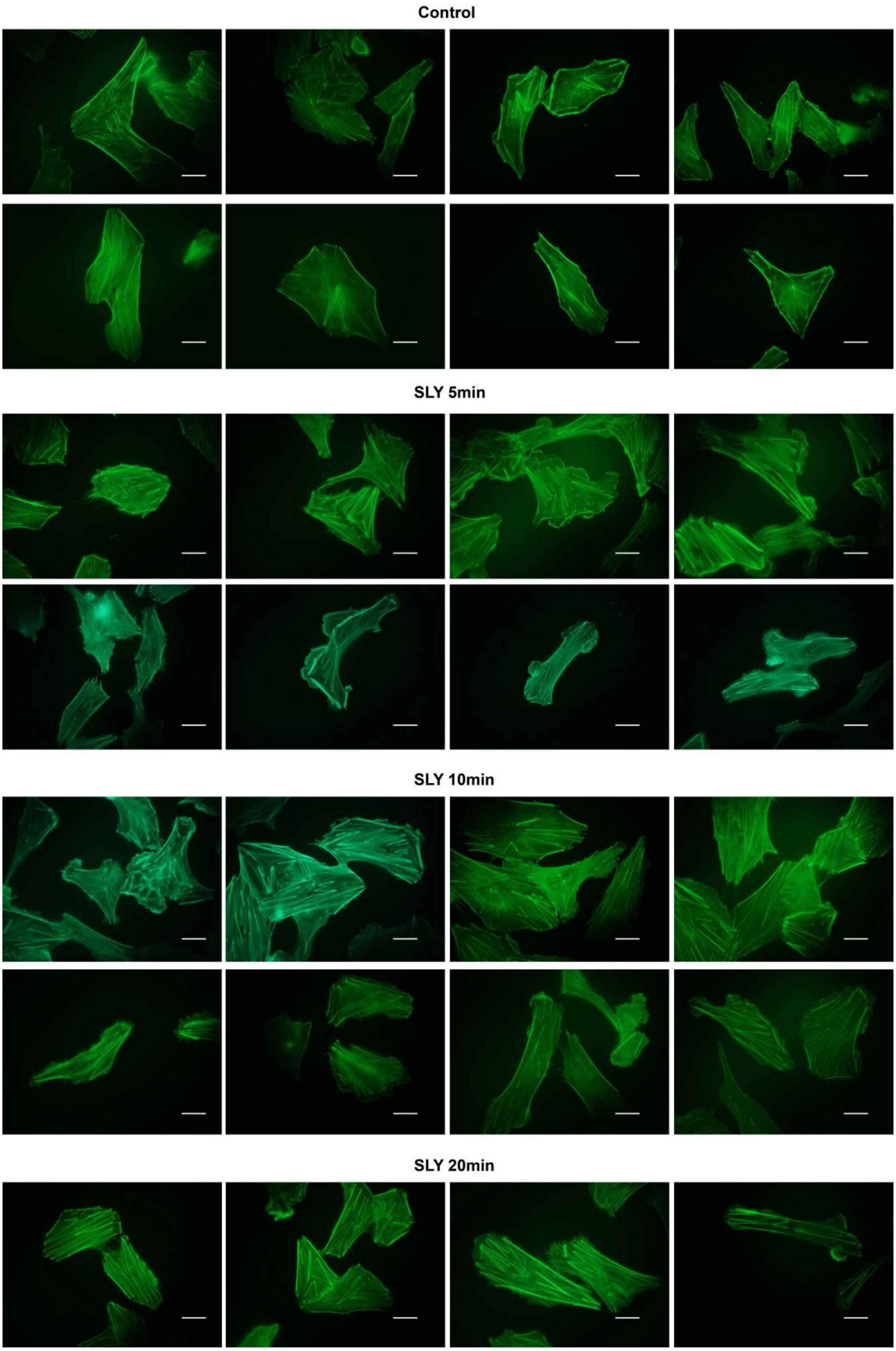

Figure S2. Analysis of suilysin-induced changes in the organization of the actin cytoskeletons of hBMEC cells. Cells were treated with sublytic concentration of suilysin protein for indicated periods.

Actin filaments were stained with FITC-labeled phalloidin.

Materials and Methods

Bacteria, cells, and culture conditions

Streptococcus suis serotype 2 strain 05ZYH33 was originally isolated from human patients in Sichuan, China. The strain was positive for muramidase-released protein (MRP), extracellular factor (EF), and suilysin (SLY). *Streptococcus suis* serotype 2 strain 1330 was originally isolated from healthy piglets in Canada. The strain was negative for MRP, EF, and SLY. Bacteria were grown overnight on sheep blood agar plates at 37 ℃ and isolated colonies were used as inocula to culture in Todd-Hewitt broth (THB) at 37 ℃ in 5% CO_2 atmosphere.

Cells of a human brain microvascular endothelial cell line, hBMECs, were immortalized by transfection with the SV40 large T antigen and retain the morphologic and functional characteristics of primary brain endothelial cells. They were successfully used in other studies to model human BBB interactions with host cells and infectious microorganisms. Here, the cells were maintained in RPMI 1640 medium containing 10% heat-inactivated fetal bovine serum (FBS), 10% Nu-Serum (BD BioSciences, Palo Alto, CA, U.S.), 2 mM glutamine, 1 mM pyruvate, penicillin (100 U/ml), streptomycin (100 μg/ml), essential amino acids, and vitamins. They were incubated at 37 ℃ in a humidified 5% CO_2 atmosphere.

Preparation of *S. suis* culture supernatant

Single colonies of *S. suis* strain was inoculated into THB medium and cultured overnight at 37 ℃ in 5% CO_2. The next day, bacteria were inoculated at 1% into new THB medium and cultured further for 5 h. Bacteria were collected and centrifuged at 10,000 × g at 4 ℃ for 10 min, the supernatant was collected and passed through a 0.2 μm sterile filter.

Preparation of suilysin protein

Natural suilysin protein was isolated and purified from the culture supernatant of *S. suis* strain 05ZYH33 by ammonium sulfate precipitation, followed by anion-exchange, and then hydrophobic chromatography. *Escherichia coli* BL21 (DE3) were induced to express histidine-tagged recombinant suilysin (wild and P353L mutant) proteins with 1 mM IPTG. They were expressed as inclusion bodies. The pellet was purified and refolded using a simple and efficient purification process and on-column refolding according to the recombinant protein purification handbook (GE Healthcare).

Hemolytic activity and cytotoxicity activity assays

The hemolytic activity of *S. suis* culture supernatant and purified suilysin proteins was determined by titration as described previously (Jacobs et al., 1994). The reciprocal of the highest dilution of a sample that exhibited at least 50% of red blood cell lysis was taken as the titer of the suilysin and expressed in hemolytic units (HU). Cytotoxic activity was determined using a CytoTox 96® Non-Radioactive Cytotoxicity Assay Kit (a lactate dehydrogenase (LDH) detection kit; Promega, Madison, WI, USA)

according to the manufacturer's instructions. Briefly, 100 μl hBMEC cells (10^4/ml) were prepared in the wells of a 96-well culture plate. Serial dilutions of each tested sample were prepared in 150 μl/well and co-incubated for 4 h in a humidified chamber at 37 ℃ and 5% CO_2. Four controls (target cell spontaneous LDH release, target cell maximum LDH release, culture medium background, and volume correction control) were produced as suggested in the manual. The plate was centrifuged at 250 × g for 4 min. Then, 50 μl aliquots of supernatant were transferred from all wells to a fresh 96-well flat-bottom plate. Reconstituted substrate mix (50 μl) was added to each well of the plate. After incubation at room temperature for 30 min, 50 μl of stop solution was added to each well, and the absorbance at 490 nmwas read and recorded.

Culture treatment

hBMEC cells were treated with suilysin at a final concentration of 0.3 μg/ml or *S. suis* culture supernatant at a final sublytic concentration for specific periods. When indicated, suilysin or *S. suis* culture supernatant were inactivated by mixing suilysin stock with water-dissolved cholesterol for 15 min. Inhibitors were applied at the following final concentrations when indicated: 10 μM ROCK inhibitor Y27632 (Sigma), 1 h before cell treatment; 2 mM MβCD (Sigma), 15 min before cell treatment.

Actin staining

hBMEC cells were seeded in complete culture medium on glass coverslips in a

12-well culture plate. Once the cells were 30–40% confluent, the medium was replaced with culture medium containing 1% FBS and cultured for another day. After serum starvation, cells were incubated in serum-free medium and treated with suilysin. After fixation in 4% paraformaldehyde in PBS, cells were permeabilized with 0.2% Triton-X 100 in PBS and incubated with FITC-phalloidin for 30 min at room temperature in the dark. Actin filaments were viewed on a fluorescence microscopy (BX51, Olympus) system by using a 100X oil immersion objective.

GTPase activity assays

GTPase activity was evaluated using Rho, Rac1, and Cdc42 activity kits (Cytoskeleton, Inc., USA) according to the manufacturer's instructions. Briefly, cells were grown under appropriate culture conditions. After treatment as described in the manual, hBMEC cells were washed once with ice-cold PBS (pH 7.2) and lysed on ice with lysis buffer (25 mM HEPES, pH 7.5, 150 mM NaCl, 1% NP-40, 10 mM $MgCl_2$, 1 mM EDTA, 2% glycerol, 2.5 mM Na_3VO_4) supplemented with phosphatase cocktail, and complete mini protease inhibitor (Roche). Cell lysates were harvested with a cell scraper and centrifuged at 10,000 × g, 4 ℃ for 2 min. Cell lysates were incubated with rhotekin-RBD beads (for evaluation of Rho activity) or PAK-PBD beads (for evaluated of Rac1 and Cdc42 activity) for 1 h at 4 ℃ under gentle rotation and then washed. Bound proteins were analyzed using western blotting. The protein signals were developed with SuperSignal West Dura Extended Duration substrate (Pierce) and imaged using an NE-1000 charge-coupled device (CCD, BGI-GBI Biotech Co., China).
